# Bio-selective hormonal breast cancer cytotoxic and antioxidant potencies of *Melia azedarach* L. wild type leaves

**DOI:** 10.1016/j.btre.2020.e00437

**Published:** 2020-02-17

**Authors:** Martha Ervina, Hadi Poerwono, Retno Widyowati, Katsuyoshi Matsunami, Sukardiman

**Affiliations:** a Doctoral Program of Pharmaceutical Sciences, Department of Pharmacognosy and Phytochemistry Faculty of Pharmacy, Universitas Airlangga, Indonesia; b Department of Pharmaceutical Biology, Faculty of Pharmacy, Widya Mandala Catholic University, Indonesia; c Department of Pharmaceutical Chemistry, Faculty of Pharmacy, Universitas Airlangga, Indonesia; d Department of Pharmacognosy and Phytochemistry, Faculty of Pharmacy, Universitas Airlangga, Indonesia; e Department of Pharmacognosy, Graduate School of Biomedical & Health Sciences, Hiroshima University, Japan

**Keywords:** Antioxidants, DPPH, FRAP, LCMS, *Melia azedarach*, TPC, T47D cytotoxic

## Abstract

*Melia azedarach* L. is used widely in traditional medicine for local or systemic ailments. Although studies exist on phytochemicals and potencies of Chinese and Indian cultivars of *Melia*, the present study investigated *in vitro* antioxidant properties of *Melia* wild type and its cytotoxicity against T47D cell. The ethanolic extract of the *Melia* leaves was fractionated with n-hexane, ethyl acetate and water, and the secondary metabolites were obtained. The antioxidant properties were determined with IC_50_ DPPH (2,2-diphenyl-1-pycrylhydrazyl) radical and FRAP (ferric reducing antioxidant power), while the cytotoxicity was determined with the MTT method. The total phenolic (TPC) and β-sitosterol (SC) contents were also measured. The results showed that the ethyl acetate fraction had higher antioxidant and cytotoxic activities (IC_50_ 211.89 ± 10.86 and 147.90 ± 8.49 μg/mL, respectively) than others. Significant (p < 0.05) correlations were observed between TPC, IC_50_DPPH, FRAP and IC_50_T47D. LC-EI MS analysis of the ethyl acetate fraction revealed the steroid and triterpenoid saponins, limonoids and quercetin glycosides, which influenced the medicinal properties of the *Melia* leaves. *Melia azedarach* L. wild type leaf extracts are a promising natural resource for managing breast cancer.

## Highlights

• 1st report on physical qualities and phytochemical content of *M*. *azedarach* wild type leaves extract and fractions.
• Ethyl acetate fraction was the most active against bio-selective hormonal breast cancer T47D cell cytotoxic and antioxidant activities.
• The phytochemicals content of active fraction was steroids and triterpene saponin, limonoid (toosendanin, meliarachin, salannin, salannal, 12-hydroxyamoorastatin, meliacarpinin and its derivates), and flavonoids (quercetin glycoside).
• Significant (p < 0.05) correlations were observed between TPC, IC_50_DPPH, FRAP and IC_50_T47D.

## Graphical abstract

**Figure:**
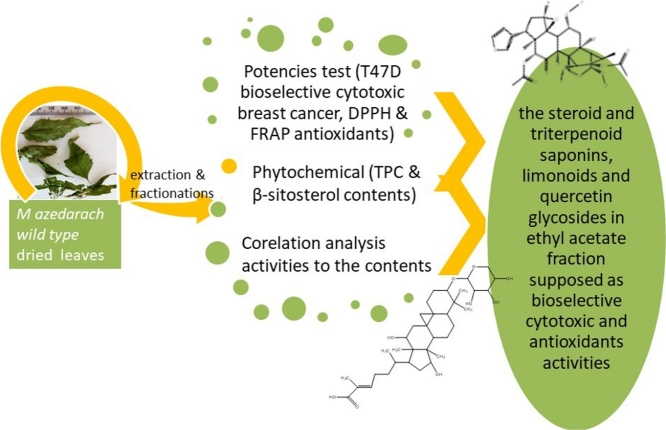

## 1 Introduction

Cancer is a global burden due to its mortality and morbidity. Its incidence and prevalence are rapidly growing worldwide. It has become a major cause of death on productive ages in all countries. Among cancer types, breast cancer is the most diagnosed and the major cause of death in females (11.6 %). The uncontrolled growth of cancer cells, resulting from genetics, infections and life styles, are complex [1]. Mutagenesis by DNA and free radical damages are related to the cancer cell initiation phase. Chemotherapies are the most widely used management technique, but they naturally kill normal and neoplastic cells. Although reactive oxygen species (ROS) play a role in cancer cell death mechanisms, they generally affect human bodies adversely. Some of the adverse ROS chemotherapies include alkyl sulfonates, ethyleneamines and hydrazines (alkylating agents), doxorubicin (anthracyclines), cisplatin and carboplatin (platinum coordination complexes), etoposides (podophyllin derivatives), and irinotecan and topotecan (camptothecins) [2]. However, intakes of antioxidant supplements, as primary and secondary steps to cancer preventions, eliminate the ROS side effects, improve tumor responses and increase patient survival rates [3].

With advances in pharmaceutical science, chemotherapy synthetic drugs are also being developed and improved to minimise these ROS side effects. Nevertheless, the array of plants available and their different phytochemicals with anticancer activities have opened research into medicinal plants to complement chemotherapy synthetic drugs for managing all types of cancer. For example, taxol from *Taxus brevifolia* (Taxaceae), vinblastine and vincristine from *Catharanthus roseus* L. G. Don (Apocynaceae), and etoposide and teniposide from *Podophyllum sp.* Linn (Berberidaceae), camptothecin, paclitaxel, homoharringtonine and many natural-derived compounds have been studied [4]. Their contents of terpenes, phenolics and alkaloids are examples of secondary metabolites with combined cytotoxic and antioxidant activities.

There are many other plants, however, that have not been studied to a comparable extent, and their medicinal potential is yet to be fully understood. One of these plants is a mahogany plant Chinaberry (*Melia azedarach* L.) that originates from Asia but now have a global spread. Three types (wild, Chinese and Indian) of *M. azedarach* are known based on the average size of the plants [5]. Almost all the parts of the Chinese and Indian types are used in traditional medicine, for example, their bark, seed, root and leaves are reported [[6], [7], [8]] to have antiparasitic, antifungal, diuretic, emmenagogue, antibacterial, antimalarial, hepatoprotection, antioxidant, antifertility and antipyretic properties or activities. Even though the wild type is mostly utilized for wood and ecological purposes, relatively, the cytotoxicity of the plant and its medicinal properties are yet to be fully established and understood, more so the efficacies of its extracts in different solvents on different cancer cells.

Zahoor et al. found the influence of different solvents (chloroform, butanol, hexane water and ethyl acetate) on the antibacterial, antioxidant and brine shrimp cytotoxicity of *M. azedarach* bark. It was found that different solvents has difference antibacterial activity, although it can be observed that ethyl acetate extract had better antioxidant and cytotoxic compare to others [8].

Furthermore, a research revealed the influence of different solvents (ethanol, petroleum ether and water) on the phytochemical content, total phenolic content (TPC) and DPPH antioxidant activity (AA) of *M. azedarach* leaves [9]. The ethanolic extract had the highest amount of phenolic compounds and exhibited the strongest antioxidant activity compared to petroleum ether and aqueous extracts. The toxicity of an ethanolic extract of the plant’s leaves against vero cells had an IC_50_ >1000 μg/mL [10]. Another study, against HT-29, A-549, MCF-7, HepG-2 and MDBK cell lines, observed that the methanolic extract of the plant’s leaves safer in term of cytotoxic activity compared to the extracts of its pulps and seeds [11]. Cytotoxic activities of the bark and root bark of *M. azedarach* against some cells have been reported and related to their steroid and limonoid tirucallane contents [[12], [13], [14], [15], [16]].

Based on the chemotaxonomy point of view, however, we are not aware of any in-depth study on the selective hormones of cytotoxic cells and antioxidant activities of *M. azedarach* leaves’ extracts and fractions, specifically on breast cancer cells. The T47D and MCF7 are hormone dependent cell lines, which are mostly used *in vitro* breast cancer cell line research. The T47D reveals more in screening breast cancer phytochemical targeting compounds though. It’s an ideal object for experimental progesterone-specific effects of breast cancer, as it is susceptible to progesterone in the presence of estrogen, while the MCF7 is not [17]. Using the wild type of *M. azedarach*, and to understand the potential activity of the plant on selective progesterone–estrogen receptor targeting breast cancer, the objectives of the study were, therefore, to investigate the cytotoxic activity against T47D cell line, antioxidant activities using DPPH and FRAP of the extracts and fractions; and examine correlations among the cytotoxic and antioxidant parameters.

## 2 Materials and methods

### 2.1 Plant material

*M. azedarach* dried leaves were obtained on dried season from Materia Medica Batu, which is an Indonesian government office on medicinal plants. Upon identification (No. 074/346/102.7/2017), the dried leaves were stored (herbarium No. Ma011017) prior to analysis [18] for drying shrinkage and ash, moisture and ethanol-soluble contents, and phytochemical screening [19] using standard procedures.

### 2.2 Preparation of the extract

The dried leaves were ground and extracted with 96 % ethanol (1:10) three times each for 24 h at room temperature. The solvent was removed in a rotary vacuum evaporator to result of the ethanolic extract (E). It was dispersed in water (1:10) and fractionated in a separating funnel with n-hexane (1:1) and ethyl acetate (1:1) to obtain n-hexane (FH), ethyl acetate (FE) and water (FW) fractions. Three replicates of the extract and fractions were done before their phytochemistry screening and analyses for cytotoxic and antioxidant activities.

### 2.3 MTT (3-(4,5-dimethylthiazole-2-yl)-2,5-diphenyl tetrazolium bromide) Cytotoxic test of T47D cell

The T47D cell lines (epithelial cells of human ductile pleural effusion from a 54-year-old mammary gland tissue) were kindly provided by Parasitology Laboratories, Faculty of Medicine, Gadjah Mada University, Yogyakarta, Indonesia. The cytotoxicity test, based on CCRC method [20], was conducted with the University’s ethics approval No. KE/FK/0310/EC/2018. The T47D cell was grown on RPMI with 10 % (v/v) Fetal Bovine Serum (FBS) and 1% (v/v) penicillin-streptomycin into 96-well plates. The optimized cell growth (70–80 % confluent) was treated with 100 μl of different concentrations of each of the extract and fractions (1000 to 10 μg/mL) and doxorubicin (100 to 0.1 μg/mL) before incubation for 24 h at 37 °C. MTT (10 %) was added to differentiate viable cells metabolism when purple formazan crystals were observed after 4 h in the dark before stopping the reactions and dissolving the crystals in 10 % dodecyl sulphate in a sulphuric acid solution. The cells were shaken (MRK-Retac) for 10 min and their absorbances were read (Elisa reader, Bio-Rad microplate reader Benchmark serial no.11565, Japan) at a wavelength of 595 nm. The absorbances were converted to percentage of viable cells as the following formula and graphed to obtain the IC_50_ (the sample concentration that inhibited 50 % of the cell growth) with linear regression analysis. Blank control (media/ A_b_) experiment was conducted. A_0_ is absorbance of cell growth without sample, while As is absorbance of cell growth treatment with sample.

% viable cells = (A_s_-A_b_)/ (A_0_-A_b_) x 100

### 2.4 DPPH (2,2-diphenyl-1-pycrylhydrazyl) antioxidant activity assay

The DPPH antioxidant activity was assayed with a Microplate reader UV/VIS spectrophotometer (Multiskan Go, Thermo Scientific, Finland) using a published procedure on the concentration range sample (100–1000 μg/mL for extract and fractions; while 1,0–30 μg/mL for rutin), and the absorbances were read at a wavelength of 517 nm [21] with a solvent blank. The IC_50_ antioxidant activity (IC_50_AA) was estimated using a linear regression analysis. Rutin was used as the reference. The formula to obtain % inhibition as A_0_ (absorbance of DPPH without sample), and As (absorbance of DPPH with samples)

% inhibition = ((A_0_-A_s_)/ A_0_) x 100

### 2.5 Ferric reducing antioxidant power (FRAP)

The FRAP was carried out according to [22], in which the colored complexes of the antioxidant samples reacted with potassium ferricyanide, trichloroacetic acid and ferric chloride, and measured at 700 nm wavelength with the spectrophotometer. The samples were prepared at 100 μg/mL, while for rutin was made on concentrations range (50–200 μg/mL). A high absorbance reflects a high antioxidant potency, and this was calculated equivalent to rutin, a natural well-known glycoside flavonoid antioxidant. Solvent and reagent blanks were also analyzed.

### 2.6 Determination of total phenolic content (TPC)

The TPC was determined with the Folin-Ciocalteau (FC) reagent with slight modifications to the micro-plate preparation [21]. The samples were prepared at 100 μg/mL; while for gallic acid was made base on pre-experiments concentrations range (10–200 μg/mL) and absorbances were measured with the reader at 765 nm wavelength using a solvent blank. The TPC (μg GAE/ mg extract or fraction) was reported as gallic acid equivalents.

### 2.7 Determination of β-sitosterol content (SC)

The determination of β-sitosterol content was done according to Sutar et al. (2014) [23] with slight modifications. The extract and fractions were made at 100 μg/mL; while for *β*-sitosterol were prepared on concentrations range (100–1500 μg/mL) and spotted 10 μL each on TLC silica gel plates. The plates were developed with n-hexane:ethyl acetate (7:3) and sprayed with anisaldehyde-SO_4_ to determine the areas of *β*-sitosterol purple spots at 517 nm wavelength (TLC Scanner Camag 3, Switzerland). The SC is reported as μg *β*-sitosterol equivalent/mg of extract or fraction and calculated from a *β*-sitosterol calibration curve.

### 2.8 LCMS (Liquid Chromatography-Mass Spectrophotometry) of the ethyl acetate fraction

The ethyl acetate fraction was pre-treated in a solid-phase extraction (Qasis® HLB solvents, Waters, Milford, USA), dissolved in methanol and filtered through a 0.2 μm syringe membrane filter before chromatographic analysis [24]. The C-18 (1.8 μm, 2.1 × 100 mm) ultra-performance liquid chromatography (Acquity UPLC®-H class system, Waters, Milford, USA) was HSS column at 50 °C). The mobile phase and flow rate were followed as described [21] so was with the electrospray ionization mass spectrometry (MSsystem (Xevo G2-S QTof, Waters, Milford, USA). The results were analyzed with MassLynx 4.1 program to determine the Rt, *m/z* fragments and molecular formula. The predictive compounds were obtained from similarities of literature of Melia’s compounds, mass bank (Fiehnlab), pubchem or chemspider.

### 2.9 Statistical analysis

The data was presented as average values and standard deviations of triplicates. The one-way ANOVA was used for mean comparisons at a 5% significant level with SPSS version 24 and the Pearson correlation analysis was also performed.

## 3 Results and discussion

### 3.1 Physical and phytochemical profile of the extract

The *M. azedarach* used was the wild type, and it usually has big trees that can be up to 40 m high. This wild type is usually different from the medium-sized Chinese and Indian types [5]. Table 1 presents the physical and phytochemical quality of the extract and the fractions of the leaves. This is the first report on the quality of *M azedarach* dried leaves’ extracts and fractions, while earlier studies were on the plant’s bark [6]. The drying shrinkage was low (1.67 ± 0.12 %), and it showed that the leaves was dried enough for further extraction processes. The ash content (6.77 ± 0.28 %) was compared to the data of the plant’s bark. Interestingly, the ethanolic soluble content (27.28 ± 1.30 %) is much higher than about 6% reported for the plant’s bark. The extract yield was about 30 %, while the yields of the fractions ranged from 8 to 65%, and there were differences in their physical colors. The extract yield in this study that used a semi-continuous repeated percolation method, is higher than the yields of 11 % [9] and 27 % [25] reported when a maceration procedure was used. However, the extract yield is comparable to a yield of about 35 % earlier reported for dried *M. azedarach* fruits [26]. Apart from differences in extractions, plant parts, and plant growth conditions (*e.g.* environments) will affect extract yields.

**Table 1:** The quality parameters of *M azedarach* dried leaves, extract and fractions*.

Parameter	Dried leaves
Identity	*Melia azedarach* leaves
Macroscopic appearance	Medium and deciduous tree, leaves is opposite non-decussate phyllotaxis with specific inipinnate (imparipinnate) compound leaves, 3–8 cm long, serrated edge, typical smelling when squeezed, dark green to pale green colour

Ethanol soluble extract content (%)	27.28 ± 1.30
Water soluble extract (%)	33.85 ± 0.56
Total ash content (%)	6.77 ± 0.28
Drying shrinkage (%)	1.67 ± 012

Parameter	Ethanolic extract (E)	Fractions		
		Hexane (FH)	Ethyl acetate (FE)	Water (FW)
Appearance	Greenish black thick extract	Dark green, oily extract	Dark green thick extract	Brownish viscous extract
Yield (%)	30.1±0.42	27.30±1.03	8.38±0.55	64.71±2.54
Tannin	+	–	–	+
Flavonoid	+	–	+	+
Saponin	+	–	+	+
Steroid	+	+	+	–
Alkaloid	+	+	+	+
Glycoside	+	–	+	+
Anthraquinone	–	–	–	–

For the yields, values with different superscripts are significantly different (p < 0.05).

+ = present, - = absent.

* E = ethanolic extract, FH = hexane fraction, FE = ethyl acetate fraction and FW = water fraction.

With respect to the yields of the fractions, ethyl acetate yielded the least, possibly because of the semi polarity characteristic of the solvent, while water yielded the highest. Both the extract and the fractions contained tannins, flavonoids, saponins, steroids, alkaloids and glycosides (Table 1), and these compounds had also been reported by Ahmad *et al.* [9]. It can be observed in Table 1 that the fractions showed differences in the phytochemical profiles.

### 3.2 Cytotoxicity and antioxidant activities

**Fig. 1:**
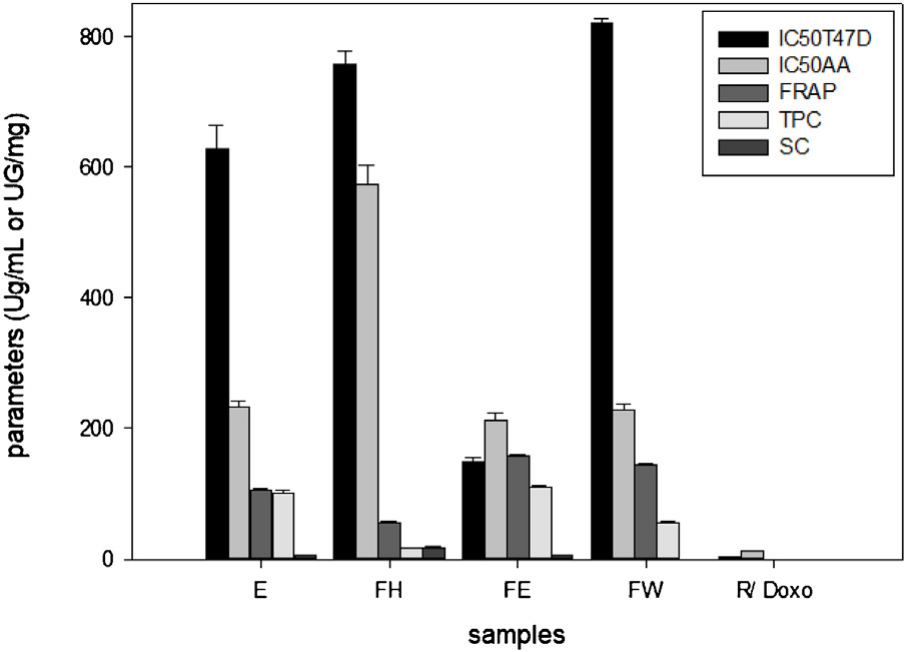
Parameters histogram of the samples result. The lower IC_50_AA and IC_50_T47D of FE showed the potent of its antioxidant and cytotoxic than others samples.

Table 2 presents the cytotoxicity (IC_50_T47D) of the samples that ranged from 148 (FE) to 820 μg/ml (FW). The IC_50_ antioxidant of the samples ranged from 212 (E) to 574 μg/ml (FH), while their FRAP was from 55 (FH) to 158 (FE) μg/mg rutin equivalent. The sequence of potential cytotoxicity was FE > E > FH and FW, while for both (DPPH and FRAP) antioxidant activities were FE > FW > E and FH (Fig. 1). The FE was effective on the T47D cells at a lower IC_50_ compared to vero cells (>1000 μg/mL) [10], though it was not as strong as doxorubicin (3.23 ± 0.22 μg/mL), the chemotherapy drug positive control. Wu et al. [27] found that three out of six cytotoxic steroids from ethyl acetate fractions of an ethanolic extract of *M. azedarach* leaves were effective against human cancer cell lines A549, H460 and U251. The three cytotoxic steroids were (20*S*)-5,24(28)-ergostadiene-3*β*,7α,16*β*,20-tetrol, (20*S*)-5-ergostene-3*β*,7α,16*β*,20-tetrol and 5-stigmastene-3,7,20-triol with an IC_50_ of about 12 to >80 μg/ml compared to IC_50_ of about 7–15 μg/mL of 5-fluorouracil. Other reported cytotoxic compounds from the plant are tirucallane triterpenes from dichloromethane and trichilin from chloroform solubles of methanolic extracts of the plant’s fruits and root barks [28,29]. Furthermore, melianones exhibited high cytotoxic effects (IC_50_ 3.6 μg/mL), while 21-*β*-acetoxymelianone and 3-*α*- tigloylmelianol were classified as having moderate antiproliferative effects (IC_50_ of 100 and 91.8 μg/mL), whereas there were no reports on cytotoxic or antiproliferative effects of methyl kulonates. Among limonoids isolated from *Melia* root bark, it was 1-cinnamoyl-3-hydroxy-11-methoxymeliacarpinin that showed significant cytotoxic activities against P388 lymphocytic leukemia (1.5 μg/mL). The structure activity revealed the influence of C-3 and C-20 acetate substituents, though 1-deoxy structures decreased the cytotoxicity. Furthermore, the trichillins had strong cytotoxic activities in a range of 0.011–5.4 μg/mL, with 12-deacetyltrichilin being the most cytotoxic substance against P388 cells [29]. These results showed the importance of purification and isolation of potential substances to enhance cytotoxic activities, especially to selected breast cancer cell lines.

**Table 2:** IC_50_T47D, IC_50_AA TPC and SC of the *M azedarach* extract and fractions*.

Samples	(μg/mL)		(μg/mg)		
	IC_50_ T47D	IC_50_ AA	FRAP (RE/ sample)	TPC (GAE/ sample)	SC (*β*SE/ sample)
E	628.05 ± 35.69^c^	232.00 ± 11.00^b^	106.20±1.53^b^	102.18 ± 2.59^c^	5.49 ± 0.37^b^
FH	757.09 ± 21.57^d^	574.25 ± 29.71^c^	55.08±2.64^a^	16.33 ± 0.23^a^	17.38 ± 2.47^c^
FE	147.90 ± 8.49^b^	211.89 ± 10.86^b^	157.75±2.51^d^	109.43 ± 3.54^d^	5.04 ± 0.12^b^
FW	820.26 ± 8.25^e^	229.32 ± 8.10^b^	144.84±2.67^c^	55.72 ± 1.36^b^	nd^a^
R	--	11.78 ± 0.74^a^	--	--	--
Doxo	3.23 ± 0.22^a^	--	--	--	

* IC_50_ = inhibition concentration, T47D = T47D cell line, AA = DPPH antioxidant activity, FRAP = ferric reducing antioxidant potency, RE = rutin equivalence, TPC = total phenolic content, GAE = gallic acid equivalence, SC = equivalent to *β*-sitosterol content, *β*SE = *β*-sitosterol equivalence, R = rutin, Doxo = doxorubicin, nd = not detectable, -- = not tested. TPC linear regression equation y = 0.0020x+0.0144, R^2^ = 0.9993; SC y = 624.32x+331.10, R^2^ = 0.9982; while rutin linearity y = 0.0009x+0.0048, R^2^=0.9803). Different superscript letters between the means in the same column, are statistically different significantly (p < 0.05).

The IC_50_AA and FRAP values of FE were the lowest (Table 2). Previous results showed young leaves exhibited higher DPPH antioxidant activities than old leaves [14], while Orhan et al. [30] found ethyl acetate extracts of the plant’s leaves and fruits to be most notable in iron and ferrous metal-chelating assays. The flavonoid quercetin-3-*O*-neohesperidoside, rutin, kaempferol-3-*O*-rutinoside and kaempferol-*O*-dihexoside were found as the main radical scavengers [14]. Other studies also found that rutin, quercetin-3-*O*-neohesperidoside and kaempferol had potent DPPH radical scavenging activities (IC_50_ of 4–6 μM) [31,32]. The chemical structures of rutin and quercetin-3-rutinoside-7-rhamnoside had glycosylation position, esterified sugar type, 2,3-double bond in conjugation with 4-oxo function in the C-ring and the twist angles of the B-ring compared to the A- and C-rings that determine its ability to scavenge free radicals [33]. It was also found that kaempferol-3-*O*-rutinoside exhibited strong antioxidant activities. Jafari et al. [11] isolated rutin, kaempferol-3-*O*-robinobioside, kaempferol-3-*O*–rutinoside, and isoquercetin (flavonol 3-*O*-glycosides) also from methanolic extracts of *M. azeadarach* leaves. The flavonols in the leaves were highly associated with their medicinal effects.

### 3.3 Total phenolic and β-sitosterol contents

The results of TPC and *β*SE linear regression equations were y = 0.0020x+0.0144, R^2^ = 0.9993 and y = 624.3201x+331.1001, R^2^ = 0.9982). The purple spot of TLC *β*-sitosterol was in a good separation (R_f_ 0.67 and R_s_ 0.98–1.2) from other compounds. Table 2 also presents the TPC (16–109 μg GAE/mg) and SC (5–17 μg *β*SE/mg) of the extract and fractions, with the latter was not detected in the FW sample. Ethanol had been shown to better extract phenolic compounds from *M. azedarach* than water and petroleum ether [9]. Other studies have shown that ethanol, ethyl acetate and water also solubilize phenolic compounds, while *β*-sitosterol is more soluble in ethyl acetate and hexane [34]. Nampoothiri et al. [35] also found that ethyl acetate fractions exhibited higher antioxidant, DPPH, and others radical (superoxide and hydroxyl) scavenging activity than hexane and water fractions.

### 3.4 The LC–MS of the ethyl acetate fraction

**Fig. 2:**
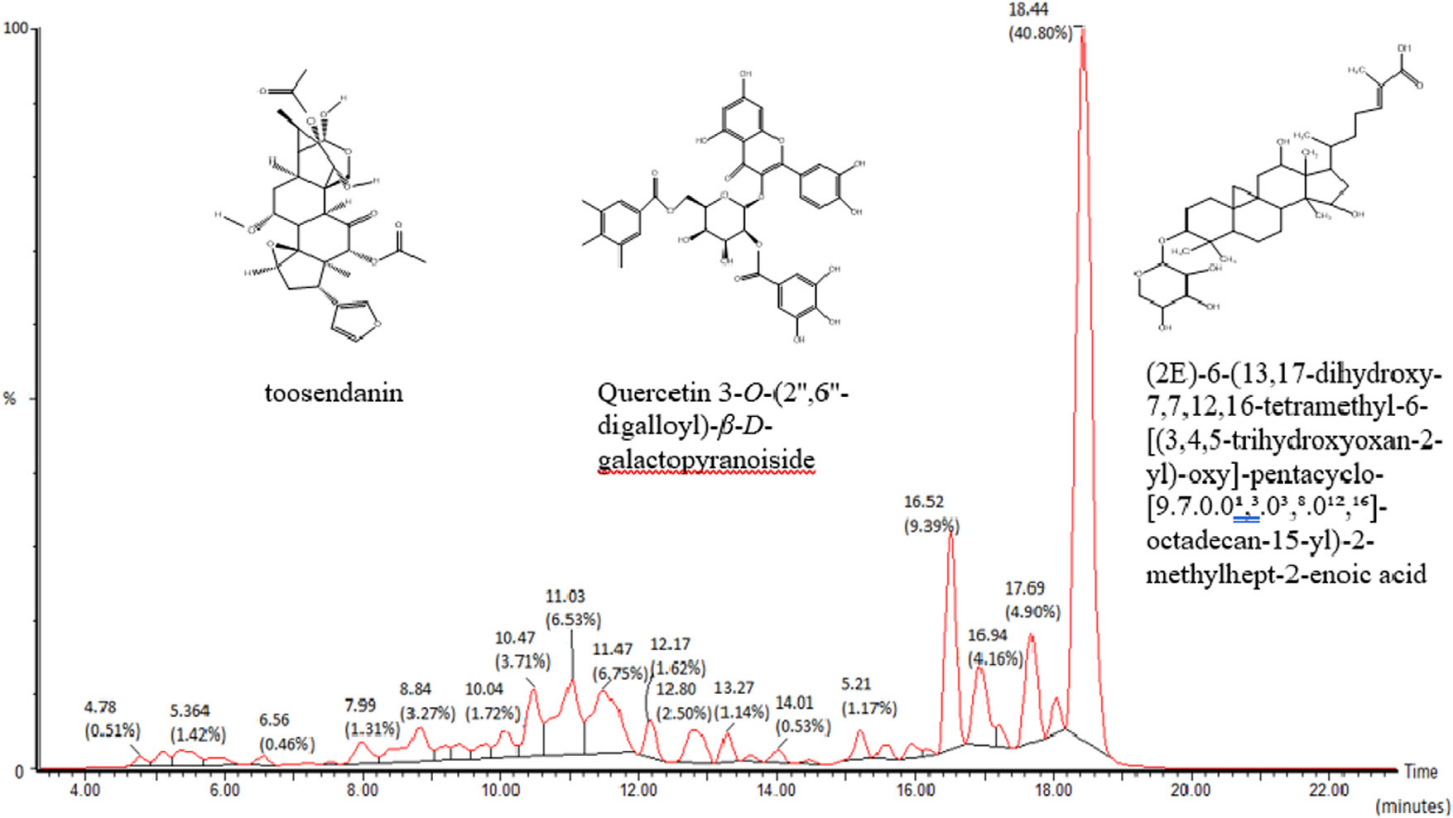
LC- EI MS chromatogram results analysis and predictive phytochemicals content from the *M. azedarach* leaves ethyl acetate fraction. It contents of limonoid (toosendanin, meliacarpinin, 12-hydroxyamoorastatin and their derivates, meliarachin, salannin and salannal), flavonoid glycosides (quercetin-7-O-*β*-d-glucopyranoside and quercetin 3-*O*-(2′',6′'-digalloyl)-*β*-d-galactopyranoiside), saponins (triterpene and steroid glycosides), and triterpene aglycone.

Being the most cytotoxic (Table 2), the FE sample was processed on to understand and identify its constituents. The LCMS had been used to predictive *Melia’s* FE fraction compounds. Though not ascertain as isolate identification, it provides more specific screening of phytochemical compounds compare to conventional method. Fig. 2 shows 32 peaks of LC separation of the FE fraction. They revealed 15 peaks with percentage area above 1% and ion fragments of the FE fraction phytocontents. The peaks were predictive as saponins (steroid glycoside), limonoids, triterpenoids and polyphenolics compounds (Table 3). The highest percentage was saponin with 40.80 % on Rt 18.438 min, while the lowest was the flavonoids with 4.68 % on Rt 5.364 and 8.841 min.

**Table 3:** Predictive compounds of FE *M. azedarach.*

Peak No	Rt (minutes)	% area	m/z [M+H]	Predictive compounds [molecular formula, M]	References
4	5.364	1.42	465	Quercetin-7-*O*-*β*-d-glucopyranoside [C_21_H_20_O_12_, 464.4]	[37,38]
8	7.987	1.31	613	Salannal [C_34_H_44_O_10_, 612.716]	[37]
9	8.841	3.27	763	Quercetin 3-*O*-(2′',6′'-digalloyl)-Beta-d-galactopyranoiside [C_38_H_34_O_17_, 762.7]	[37]
13	10.041	1.72	573	Meliarachin B [C_30_H_36_O_11_, 572.6]	[37]
14	10.474	3.71	683	(2*R*,3*S*,4*S*,5*R*,6*S*)-3,4-dihydroxy-6-[3,7,8-trihydroxy-2-(4-hydroxy-3-methoxyphenyl)-4-oxochromen-5-yl]-oxy-5-[(2*S*,3*R*,4*R*,5*R*,6*S*)-3,4,5-trihydroxy-6-methyloxan-2-yl]-oxyoxan-2-yl]-methyl acetate [C_30_H_34_O_18_, 682.1745]	[36]
15	11.032	6.53	575	Toosendanin [C_30_H_38_O_11_, 574.616]	[15,39]
16	11.474	6.75	697	Methyl (23*S*)-7,14,23-trihydroxy-4-methoxy-6,16,22-trimethyl-25-[(*E*)-3-phenylprop-2-enoyl]-oxy-3,9,11,17,20-pentaoxaoctacyclo [17.6.1.18,15.01,5.06,18.07,16.010,14.022,26]-heptacos-12-ene-4-carboxylate (1-cinnamoyl-3-hydroxy-11-methoxymeliacarpinin) [C_30_H_49_O_18_, 696.7]	[37]
17	12.169	1.62	557,497	toosendanin derivates [M–H_2_O+H]+ at 557.4 and [M–CH_3_COO+H]+ at 497.2	[15,39]
18	12.802	2.50	537	Cyclopenta[c]pyran-4-carboxylic acid, 1-(*β*-d-glucopyranosyloxy)-1,4a,5,6,7,7a-hexahydro-5-hydroxy-7-methyl-6-[(2*E*)-1-oxo-3-phenyl-2-propen-1-yl]-oxy- methyl ester, [C_26_H_32_O_12_, 536.1894]	[36]
19	13.265	1.14	597	Salannin [C_34_H_44_O_9_, 596.7]	[37,41]
23	15.214	1.17	547	Meliarachin H/I [C_29_H_38_O_10_, 546.6]	[37]
27	16.521	9.39	593, 533	12-hydroxyamoorastatin – acetyl derivate [C_30_H_40_O_12_, 592.23]	[37,40]
28	16.942	4.16	533	12-hydroxyamoorastatin [C_28_H_36_O_10_, 532.2308]	[37,40]
30	17.68	4.90	637	5-[17-(5,6-dihydroxy-6-methylheptan-2-yl)-3,12-dihydroxy-4,4,10,13,14-pentamethyl-2,3,5,6,7,11,12,15,16,17-decahydro-1H-cyclopenta[a]phenanthren-2-yl]-oxy-3-hydroxy-3-methyl-5-oxopentanoic acid [C_36_H_60_O_9_, 636.4237]	[36]
32	18.438	40.80	621	(2E)-6-(13,17-dihydroxy-7,7,12,16-tetramethyl-6-[(3,4,5-trihydroxyoxan-2-yl)-oxy]-pentacyclo-[9.7.0.0¹,³.0³,⁸.0¹²,¹⁶]-octadecan-15-yl)-2-methylhept-2-enoic acid [C_35_H_56_O_9_, 620.3924]	[36]

The quercetin was obtained with m/z [M+H] 303. Flavonoid quercetin-7-*O*-*β*-d-glucopyranoside was obtained with ion parents 465 compare to its data on [M] 464.6 [36]. The compounds on Rt 8.841 min had similar fragmentation pattern, but higher *m/z value* on 763 which was assumed as quercetin-3-*O*-(2″,6″-digalloyl)-*β*-d-galactopyranoside [M] 762.7 [[36], [37], [38]]. The MS result can not differentiate C3 or C7 bond glycoside on the aglycone flavonoid, accordingly though. Two peaks revealed toosendanin and its derivate, which have *m/z* at 557 (R_t_ 11.032 min, 6.53 % and 12.169 min, 1.62 %) [15,39]. It was also observed the ions fragmentation at *m*/z 497, 479, 437, and 419; which were identified as existence of two acetoxyl and two hydroxyl groups in toosendanin as the fragments of [M+H–H_2_O − CH_3_COOH]+, [M+H−2H_2_O − CH_3_COOH]+, [M+H–H_2_O−2CH_3_COOH]+, and [M+H−2H_2_O−2CH_3_COOH]+, respectively [39]. The 1-cinnamoyl-3-hydroxy-11-methoxymeliacarpinin with m/z [M+H] 697 was observed on Rt 11.474 min with 6.53 % [37,38]. The 12-hydroxyamoorastatin and its acetyl derivate were on Rt 16.942 and 16.521min with 4.16 % and 9.39 % respectively [40]. Highly percentage of steroids and terpen saponins compounds were detected on 18.438 min (40.80 %) and Rt 12.802 (2.50 %), while triterpenoids aglycone was on Rt 17.68 min (4.90 %). Sterol fragment was observed at R_t_ 18.438 min (*m/z* of 275 with (M+-C_3_H_7_O)18-CH_3_+side chain) with 16-ketosteroids cleavage, which was identified as *β-*sitosterol glucoside [M+H] 577 compound [12,36,37]. Others compounds were analyzed as meliarachin, salannin, and salannal as listed in Table 3. Salannin and salannal (R_t_ 13.265 min, 1.14 %; 7.987 min, 1.31 %) [41], and meliacarpinin derivates (R_t_ 11.474 min, 6.35 %) [37,42,43]. The identification process based on comparison with the most identical fragments to available references data, though others may have similarity only some part to quite different. The varieties on the technical analysis method and the limitation of the data were leading to the used of references available on the experiment or the importance of isolation to identification of the substances further. The environment influence and local variety of the *M. azedarach* wild type provide opportunity to obtain new or modified of its chemical entities. For example quercetin glycoside at Rt 8.841 min was found have longer glycoside and not identical with the references available. Others of saponin and triterpen at Rt 18.438, 17.680 and 12.802 min were identical with references data [[36], [37], [38]], but had not been reported on the Melia’s content yet. Some compounds resulted specific R_t_ and fragmentations ion pattern such as meliatoxin, meliarachin, *β-*sitosterol and flavonoids, which were suggested to be used as biomarker for *Melia* extracts [12].

### 3.5 Correlation analysis among activities and contents

Correlation analysis between the activity parameters revealed negative and positive significant outcomes. TPC significantly (p < 0.05) correlated with parameters IC_50_AA (-0.845), FRAP (0.695) and IC_50_T47D (-0.709), while IC_50_T47D significantly correlated with IC_50_AA (0.671), but there were no significant correlations between SC and others. Though they have small percentage in the fraction, phenolic compounds play important roles in cytotoxicity and antioxidant activities. The significant correlations reported were consistent with these relationships. Positive correlations had been reported for pomegranate cytotoxicity and TPC on MDA-MB-231 (0.980); and total flavonoid content to HT-29 cell line cytotoxic (0.864) [44]. Grigalius and Petrikaite [45] suggested the structure-activity relationships of both antioxidant and anticancer activities were due to ortho-dihydroxy group in ring C of flavonoids. The ortho-dihydroxy was obtained (about 4.68 %) in FE as quercetin glycoside (Table 3). Quercetin was found as powerfull hypochlorous acid, chloramines, nitric oxide, and superoxide scavengers; and also cytotoxic *to* red blood cell haemolysis, compared than that of kaempferol and isoquercitrin [46]. Quercetin significantly inhibited human breast cancer cells (MCF-7 and MDA-MB231), and moreover it mentioned has the cytoprotective role against oxidative stress through antioxidant effect, motivating apoptotic cell death *via* prooxidant activity, and inhibiting tumourigenesis [47].

Furthermore, Ashraf et al. [48] found that steroids and triterpene saponins were cytotoxic against MCF-7. Podolak et al. [49] highlighted the potential of saponins as anticancer. He found the important factors responsible for improving the cytotoxicity including structural feature, number and the sequence of sugar residues in a carbohydrate chain, also the position of sugar attachment to the aglycone. The cytotoxicity was enhance with the prolonged of the sugars chain. The saponins were stimulate apoptotic process in tumor cells, in intrinsic pathway mostly. Non apoptotic processes were also involved as cell cycle arrestment, autophagic cell, death stimulation, inhibiting of metastasis and cytoskeleton, including disintegration of the cell. Saponins are also promising as inhibiting tumor cells angiogenesis and recombinant protein toxins. Furthermore saponins have physiologically binding to nuclear receptors activity, including to conventional steroid hormone receptors (estrogen receptor, glucocorticoid receptor, mineralocorticoid receptor, and androgen receptor) and the orphan receptors (peroxisome proliferatoractivated receptor (PPAR), liver X receptor (LXR), farnesoid X receptor (FXR), and Pregnane X receptor (PXR)) [50].

Akhihisa et al. [51] revealed limonoid trichillin type of meliarachin C and salannin type of 3-*O*-deacetyl-40-demethyl-28-oxosalannin are cytotoxic to HL-60 cells by inducing apoptotic cell death. Zhou et al. [43] observed limonoids tetracyclic sendanin, trichillin and C-*seco* limonoid types with 14,15-epoxide ring and a C-19/C-29 acetal bridge exhibit very strong and strong cytotoxicity antiproliferation against Hela S3 (human epithelial cancer) cell line and against P388 cells. Yadav et al. [52] found the role of neem limonoids in mitochondria oxidative phosphorylation complexes, though not effective to p53 and Bax-independent. Neem limonoids are, however, useful for multiple cancers, including cancer-drug-resistant ones and as a novel for solid cancer therapy.

## 4 Conclusions

The ethyl acetate fraction from the *M. azedarach* L. wild type leaves ethanolic extract showed the most T47D bio-selective hormonal cytotoxicity and antioxidant activities. Significant correlations among TPC, IC_50_T47D, IC_50_AA and FRAP activities were obtained to lend credence to compound-antioxidant-cytotoxicity relationships. The ethyl acetate fraction contain steroid and triterpenoid saponins, triterpenoid, ortho-dihydroxy flavonols of quercetin glycosides, limonoid toosendanin and its derivate, 12-hydroxyamoorastatin and its acetyl derivate, 1-cinnamoyl-3-hydroxy-11-methoxymeliacarpinin, meliarachin, salannin and salannal. The compounds were potent against the breast cancer cells and showed promises in breast cancer managements.

## Ethical statement for biotechnology reports

Hereby, I /insert author name/ consciously assure that for the manuscript /insert title/ the following is fulfilled:

1) This material is the authors' own original work, which has not been previously published elsewhere.
2) The paper is not currently being considered for publication elsewhere.
3) The paper reflects the authors' own research and analysis in a truthful and complete manner.
4) The paper properly credits the meaningful contributions of co-authors and co-researchers.
5) The results are appropriately placed in the context of prior and existing research.
6) All sources used are properly disclosed (correct citation). Literally copying of text must be indicated as such by using quotation marks and giving proper reference.
7) All authors have been personally and actively involved in substantial work leading to the paper, and will take public responsibility for its content.

The violation of the Ethical Statement rules may result in severe consequences.

To verify originality, your article may be checked by the originality detection software iThenticate. See also http://www.elsevier.com/editors/plagdetect.

I agree with the above statements and declare that this submission follows the policies of Solid State Ionics as outlined in the Guide for Authors and in the Ethical Statement.

## Declaration of Competing Interest

The authors whose names are listed immediately below certify that they have NO affiliations with or involvement in any organization or entity with any financial interest (such as honoraria; educational grants; participation in speakers’ bureaus; membership, employment, consultancies, stock ownership, or other equity interest; and expert testimony or patent-licensing arrangements), or non-financial interest (such as personal or professional relationships, affiliations, knowledge or beliefs) in the subject matter or materials discussed in this manuscript.
